# Establishment of *Myotis myotis* Cell Lines - Model for Investigation of Host-Pathogen Interaction in a Natural Host for Emerging Viruses

**DOI:** 10.1371/journal.pone.0109795

**Published:** 2014-10-08

**Authors:** Xiaocui He, Tomáš Korytář, Yaqing Zhu, Jiří Pikula, Hana Bandouchova, Jan Zukal, Bernd Köllner

**Affiliations:** 1 Institute of Immunology, Friedrich-Loeffler-Institute (FLI), Federal Research Institute for Animal Health, Greifswald- Insel Riems, Germany; 2 Department of Ecology and Diseases of Game, Fish and Bees, Faculty of Veterinary Hygiene and Ecology, University of Veterinary and Pharmaceutical Sciences Brno, Brno, Czech Republic; 3 Institute of Vertebrate Biology, Academy of Sciences of the Czech Republic, Brno, Czech Republic; 4 Department of Botany and Zoology, Masaryk University, Brno, Czech Republic; Thomas Jefferson University, United States of America

## Abstract

Bats are found to be the natural reservoirs for many emerging viruses. In most cases, severe clinical signs caused by such virus infections are normally not seen in bats. This indicates differences in the virus-host interactions and underlines the necessity to develop natural host related models to study these phenomena. Due to the strict protection of European bat species, immortalized cell lines are the only alternative to investigate the innate anti-virus immune mechanisms. Here, we report about the establishment and functional characterization of *Myotis myotis* derived cell lines from different tissues: brain (*Mm*Br), tonsil (*Mm*To), peritoneal cavity (*Mm*Pca), nasal epithelium (*Mm*Nep) and nervus olfactorius (*Mm*Nol) after immortalization by SV 40 large T antigen. The usefulness of these cell lines to study antiviral responses has been confirmed by analysis of their susceptibility to lyssavirus infection and the mRNA patterns of immune-relevant genes after poly I:C stimulation. Performed experiments indicated varying susceptibility to lyssavirus infection with *Mm*Br being considerably less susceptible than the other cell lines. Further investigation demonstrated a strong activation of interferon mediated antiviral response in *Mm*Br contributing to its resistance. The pattern recognition receptors: RIG-I and MDA5 were highly up-regulated during rabies virus infection in *Mm*Br, suggesting their involvement in promotion of antiviral responses. The presence of CD14 and CD68 in *Mm*Br suggested *Mm*Br cells are microglia-like cells which play a key role in host defense against infections in the central nervous system (CNS). Thus the expression pattern of *Mm*Br combined with the observed limitation of lyssavirus replication underpin a protective mechanism of the CNS controlling the lyssavirus infection. Overall, the established cell lines are important tools to analyze antiviral innate immunity in *M. myotis* against neurotropic virus infections and present a valuable tool for a broad spectrum of future investigations in cellular biology of *M. myotis*.

## Introduction

Bats belong to one of the most abundant, diverse and widely distributed mammalian groups. In the order of *Chiroptera* which is divided into two suborders *Megachiroptera* and *Microchiroptera*, a total of 1,240 species have been yet described [1]. Bats evolved early and changed very little over the past 52 million years [2]. Their wide distribution and migratory behaviour favour bats as vectors for viruses and raise concerns over their role in zoonotic diseases [3]–[5]. Among the large number of viruses detected in bats, some like Hendra virus, Nipah virus, severe acute respiratory syndrome coronavirus (SARS), Ebola virus, West Nile virus were reported to be zoonotic [4], [6]–[11]. Also 13 of the 15 lyssaviruses, except Mokola virus and Ikoma lyssavirus, were detected in bats. In North America bats host RABV, whereas in Europe European Bat lyssavirus type 1 and 2 (EBLV-1 and EBLV-2) are found in different bat species [12], [13]. Annually, there are approximately 55,000 human deaths caused by rabies, especially in the developing countries of Asia and Africa [14]. Despite most human rabies deaths are associated with dog rabies, some of them can be directly linked to the contact with bats, such as 8 out of 226 human rabies cases were of bat origin in the Americas in 1983 and a few human cases caused by EBLVs were reported in Europe to date [15]–[18]. Although bat associated viruses can cause severe diseases in various mammals, they seem to be less pathogenic for bats [3], [19]–[25]. After experimental infection with Hendra or Nipah virus, bats showed no clinical disease, while guinea pigs succumbed to the same dose of virus [21], [22]. Similar situation was also observed in Hendra virus infection in horses and bats [24]. Lyssaviruses are the only viruses that were reported to cause clinical disease in bats [26]. However, only a small proportion of bats develop clinical symptoms after experimental infection [25], [27]. This indicates a critical difference in the development of viral disease between bats and other mammals and requires profound investigation of bat immunology and host-virus interactions.

Since all of 52 identified European bats species are endangered and strictly protected, the use of animal trials for the investigation of immune mechanisms in bats is not possible. Thus, development of stable cell lines for *in vitro* studies derived from European bat species is desirable. So far, several bat cell lines were reported in previous studies, but most of them were established from non-European bats, like Tb1-Lu from *Tadarida brasiliensis*, Mvi/It from *Myotis velifer incautus*, and several primary immortalized cell lines from *Pteropus alecto*, *Carollia perspicillata*, *Eidolon helvum* and *Rousettus aegyptiacus* [28]–[31]. Viral infection studies have been carried out in the fruit bat cell lines to investigate the susceptibility, infection kinetics of henipavirus as well as the host innate immunity [28], [32]. However, the susceptibility to lyssavirus has not yet been examined in these cell lines. Additionally, except for a brain cell line from *Eptesicus serotinus* employed to investigate the type I interferon (IFN) response after lyssavirus infection [33], the use of a bat cell line as a tool for studies into lyssavirus infection in its natural reservoir host is rare. A broader variety of bat cell lines, particularly European bat cell lines from tissues of immune relevance, is therefore urgently in demand for lyssavirus-host studies.

In this study, we established different cell lines from the European bat *M. myotis*, evaluated their susceptibility to EBLV-1, EBLV-2 and RABV infection and investigated innate immune gene responses after the polyinosinic:polycytidylic acid (poly I:C) stimulation. The established *M. myotis* cell lines present a valuable *in vitro* model to study the interactions between lyssaviruses and their natural host, and to shed light on the mechanisms of resistance in bat's central nervous system (CNS).

## Materials and Methods

### Ethics statement

Ethical approval for all of the capturing and sampling were confirmed by the competent authorities in the respective Federal Republic of Germany and Czech Republic. The Czech Academy of Sciences Ethics Committee reviewed and approved the animal use protocol No. 169/2011 in compliance with Law No. 312/2008 on Protection of Animals against Cruelty adopted by the Parliament of the Czech Republic. The capture and sampling of a *M. myotis* specimen in the Moravian Karst in November 2012 was in compliance with Law No. 114/1992 on Nature and Landscape Protection, and was based on permit 01662/MK/2012S/00775/MK/2012 issued by the Nature Conservation Agency of the Czech Republic. Established *M. myotis* cell lines from the single sacrificed specimen have been used to examine bat responses to the infection by *Pseudogymnoascus destructans* (un-published data) as well as for the present study of rabies. Three co-authors of the present manuscript concerning establishment of *M. myotis* cell lines to investigate lyssavirus infection, i.e. Hana Bandouchova, Jiri Pikula, and Jan Zukal, examine white-nose syndrome in the Czech Republic and hold the necessary permits. A paper based on these permits and excemption from Law No. 114/1992 on Nature and Landscape Protection of the Czech Republic allowing euthanasia of up to 10 *M. myotis* bats has already been published [34].

### Primary cell culture and immortalization

A single *M. myotis* male was captured in Sloupsko-Sosuvske caves of the Moravian Karst (Czech Republic, coordinates 49° 24′ 40.88″ and 16° 44′ 20.54″). The bat was kept to minimize stress and handling between capture and euthanasia in a clean plastic box with soft mesh to enable roosting under temperature of hibernation torpor of 6°C and transferred to our laboratory at Veterinary and Pharmaceutical Sciences Brno (Czech Republic) within a day. It was anesthetized to insensitiveness using isofluranum (Isofluran, Piramal Healthcare, UK), and then euthanized by decapitation and subjected to necropsy in order to collect organs and tissues. Tissues were freshly isolated from the euthanized bat, and then minced and cultured in Dulbecco's Modified Eagle Medium (DMEM) supplemented with 10% fetal calf serum (FCS), penicillin 100 units/mL and streptomycin 100 mg/mL (Sigma). Primary cells were cultured in 6-well plates till the confluence reaches 50–70%. Immortalization was done by transfection of pRSVAg1 plasmid expressing Simian Vacuolating Virus 40 large T antigen (SV40T) with lipofectamine 2000 according to the protocol (Invitrogen). Immortalized cells were expanded and stock frozen. After several passages, the mRNA expression of SV40T (in the established lines) was tested by reverse transcription PCR (RT-PCR) using SV40T specific primers [35]. The protein expression was controlled by the immunofluorescence and western blot as described below. Briefly, cells were first fixed with 3% paraformaldehyde and permeabilized with 0.5% triton X. After washing with PBS, cells were stained with mouse anti-SV40T monoclonal antibody (Santa Cruz Biotechnology) and goat anti-mouse IgG Alexa Fluor (Invitrogen) as second antibody and visualized by fluorescence microscope. For western blot, the same mouse antibody was used as primary antibody and bound antibody was detected with goat anti-mouse IgG peroxidase (Sigma). Images were developed using the ECL kit (Thermo Scientific Pierce) according to the manufacturer's instructions.

### Species confirmation of different cell lines by PCR

To confirm the identity of the established *M. myotis* cell lines derived from brain (cerebrum) (designated *Mm*Br), tonsil (*Mm*To), peritoneal cavity (*Mm*Pca), nasal epithelium (*Mm*Nep) and nervus olfactorius (*Mm*Nol), a *M. myotis*-specific PCR was developed. An NADH dehydrogenase subunit 1 (ND1) gene (Genbank accession number: DQ915043) from *M. myotis* was used as a species specific molecular marker. The genomic DNA from different cell lines was isolated by DNeasy Blood & Tissue Kit (Qiagen). The concentration and purity of genomic DNA were determined by Nanodrop (Thermo). PCR was performed using a specific primer pair ND1-F and ND1-R (Table 1) and genomic DNA as a template by GoTaq Flexi DNA Polymerase (Promega) to get the ND1 fragments. PCR products were cloned into PCR2.1 vector (Invitrogen) and transformed into *E. coli* competent cells. Plasmids were extracted from positive clones and sequenced by Applied Biosystems 3130 Genetic Analyzer (Life Technologies) at the Friedrich-Loeffler-Institute, Germany.

**Table 1:** Primers used in this study.

Name*	Sequence (5′→3′)
^a^SV40T-F	GGGTCTTCTACCTTTCTCTTCTTT
^a^SV40T-R	GCAGTGGTGGAATGCCTTT
ND1-F	TATTAGCCCTATCAAGTTTAGC
ND1-R	GGATGCTCGGACCCATAA
β-actin-F	GCGCAAGTACTCTGTGTGGA
β-actin-R	ATCTCGTTTTCTGCGCAAGT
^a^ Mx1-F	TCTACTGCCAAGACCAAGCGT
^a^ Mx1-R	CGAGGGAGCAAGTCAAAGGA
^a^ IFIT3-F	AGCAGAGGAGCTTGCAGAAG
^a^ IFIT3-R	CCGGAAAGCCATAAACAAGA
^a^ ISG56-F	CAGGCTAAATCCAGAAGATG
^a^ ISG56-R	TTCCAGAGCAAATTCAAAAT
ISG43-F	CATGATGCTGCTCAACTCTA
ISG43-R	TAAGGTGGATTGTCAAGGTC
TLR3-F	TCTCGCTCCTTCTATGGG
TLR3-R	TGCCTGGAAAGTTGTTATCG
RIG-1-F	GAAGAGCAAGAGGTAGCAAA
RIG-1-R	CCTTTGCTTTCTTCTCAAAA
MDA5-F	TCCGAATGATTGATGCCTAT
MDA5-R	ATTATCCCTCTTGCTGACCC
CD14-F	GCTCTCTTAACCTGTCCTCCG
CD14-R	CTCTGTTCAGCCGGTTGTTG
CD68-F	GCCCTGGTGCTTGTTATCCT
CD68-R	GAGGCAGCTGAGTGGTTCAG
^a^ EBLV1-F	GAAAGGKGACAAGATAACACC
^a^ EBLV1-R	ARAGAAGAAGTCCAACCAGAG
^a^ EBLV2-F	GGTGTCTGTAAAGCCAGAAG
^a^ EBLV2-R	TTATAAGCTCTGTTCAAG
^a^ RABV-F	GATCCTGATGAYGTATGTTCCTA
^a^ RABV-R	GATTCCGTAGCTRGTCCA

* F indicates forward primer, R indicates reverse primer. ^a^Primers are from previous studies [33], [35].

### Poly I:C stimulation

To evaluate the IFN response of *M. myotis* cell lines and the induction of IFN mediated signaling, poly I:C was used to stimulate the cells. Different cell lines were seeded in 24-well plates at a density ranging from 1.2 to 2×10^5^ cells/well, and cultured as described above. Around 20 hours after seeding, cells were transfected with poly I:C (Sigma) at a concentration of 10 µg/mL by lipofectamine 2000 (Invitrogen) following the manufacturer's instructions. Twenty four hours post stimulation, cells were harvested into RLT buffer (Qiagen) for RNA extraction by an RNeasy mini kit (Qiagen).

### Lyssaviruses infection

Early after immortalization, the third passage immortalized cell lines were used to check the infectivity of RABV. Cells were infected with RABV (European fox isolate, fused with green fluorescent protein, GFP) at a MOI of 10. Twenty four hours post infection (hpi), infected cells were fixed and permeabilized as described above and visualized by fluorescence microscope. *Mm*Br and *Mm*To cells that were infected with a serial MOI of 0.01, 0.1, and 1.0 were harvested at 24 hpi and used for RNA extraction. To confirm the infectivity in later passaged cells, different immortalized cell lines of more than 15 passages were infected with lyssaviruses RABV, EBLV-1 (*E. serotinus* isolate) and EBLV-2 (*M. daubentonii* isolate) at a MOI of 0.1. The infected cells were cultured as described above. Cells were collected for RNA extraction at 24 hpi and quantitative real-time PCR (qRT-PCR) was performed on the CFX96 TouchDetection System (Bio-Rad) using SensiFAST SYBR one-step kit (Bioline) according to the protocol. Immunofluorescence analysis was performed on fixed cells using FITC conjugated anti-rabies monoclonal antibody (SIFIN) at 72 hpi as described before [36]. To further confirm the susceptibility, *Mm*Br and *Mm*Nol cell lines were infected with EBLV-1 at a MOI of 0.01 to set the sensitivity at a Ct value of 22 for the inoculation dose. The viral supernatant was either changed or not changed with fresh medium at 1 hpi, and viral replication levels were measured by qRT-PCR over 72 hpi.

### Quantitative real-time PCR

qRT-PCR was introduced to measure the mRNA expression levels of immune related molecules in response to poly I:C stimulation and virus infection. The selected molecules include IFN induced genes: IFN stimulated gene 56 (ISG56), ISG43, myxovirus resistance 1 (Mx1) and IFN induced protein with tetratricopeptide repeats 3 (IFIT3), and pattern recognition receptors (PRRs): toll-like receptor 3 (TLR3), retinoic acid-inducible gene 1 (RIG-1) and melanoma differentiation-associated protein 5 (MDA+5). All of these primers were designed based the sequence resources from our own un-published sequence database and public databases of bat species. The softwares for primers design include primer premier 5, online tools: http://bioinfo.ut.ee/primer3-0.4.0/ and http://www.ncbi.nlm.nih.gov/tools/primer-blast/. Primers of target genes and internal control β-actin were listed in Table 1. qRT-PCR was performed on the CFX96 TouchDetection System (Bio-Rad) using SensiFAST SYBR one-step kit (Bioline) according to the protocol. To assess the specificity of the PCR amplification, a melting curve analysis was performed at the end of the reaction. The relative expression levels of targets were calculated by 2^−ΔΔCt^ method [37].

Molecular characterization of the *Mm*BrBecause the *Mm*Br is derived from the CNS, the target of fatal infections by lyssaviruses, a further characterization of cell type of *Mm*Br is desired to improve the understanding of the antiviral defense in the CNS. The expressions of cluster of differentiation (CD) 68, a marker for cells of macrophage lineage [38], and CD14, a marker expressed in activated microglia [39], were investigated by RT-PCR in different cell lines. Specific primers for CD14, CD68 and internal control β-actin were listed in Table 1. The RT-PCR was prepared according to the instructions of the one-step RT-PCR kit (Qiagen).

### Statistical analysis

All data were presented as means ± S.D. Statistical significant differences were analysed by one-way ANOVA using the SPSS software package.

## Results

### Permanent cell lines of different origin could be established after immortalisation

**Figure 1:**
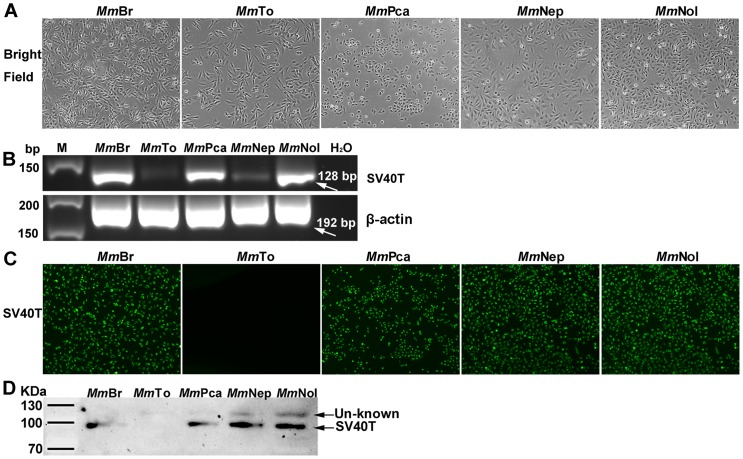
Newly established immortalized *M. myotis* cell lines from different tissues *Mm*Br - brain; *Mm*To - tonsil; *Mm*Pca - peritoneal cavity; *Mm*Nep - nasal epithelium; *Mm*Nol - nervus olfactorius. (**A**) Morphology of a 24 h culture of immortalized *M. myotis* cell lines. (**B**) Expression of SV40T transcripts in different *M. myotis* cell lines. Note the very low expression in *Mm*To. (**C**) Expression of SV40T protein visualized by immunofluorescence and (**D**) by western blot using anti-SV40T monoclonal antibody. Note the absence of SV40T protein in *Mm*To cell line.

Five *M. myotis* cell lines brain (*Mm*Br), tonsil (*Mm*To), peritoneal cavity (*Mm*Pca), nasal epithelium (*Mm*Nep) and nervus olfactorius (*Mm*Nol) were successfully established by transformation with SV40T gene integrating into the chromosomal DNA. Varying cell morphologies were observed in the cell lines, with *Mm*Br, *Mm*To, *Mm*Nep and *Mm*Nol being fibroblastic-like, and *Mm*Pca being epithelial-like (Fig. 1A). The mRNA expression of SV40T antigen was detected in all cell lines (Fig. 1B). Protein level expression was confirmed in four of the five cell lines by immunofluorescence microscopy and western blot, respectively (Fig. 1C and D). In *Mm*To, the protein level of SV40 T antigen was under detectable because the transcriptional level is significantly low determined by RT-PCR (Fig. 1B). After immortalization, all five cell lines grew for more than 30 passages. The identity of the cell lines was validated by a *M. myotis* specific PCR using ND1 gene as a molecular marker. A predicted 515-bp fragment was obtained from genomic DNA of each cell line, and further confirmed by sequencing.

### *M. myotis* permanent cell lines express major innate immune molecules

**Figure 2:**
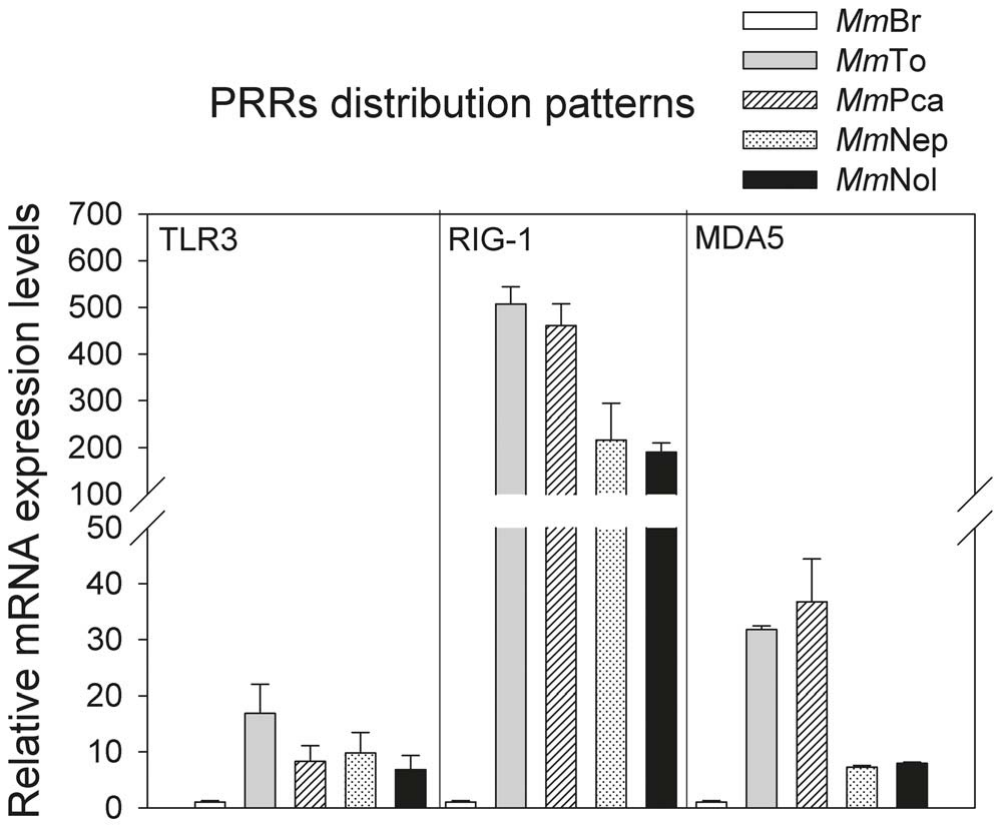
PRRs distribution patterns in the established unstimulated *M. myotis* cell lines. The mRNA expression levels of TLR3, RIG-1 and MDA5 in *Mm*Br, *Mm*To, *Mm*Pca, *Mm*Nep and *Mm*Nol were determined by qRT-PCR (n = 3). The expression level was shown as a related fold and normalized against β-actin. The expression level of different genes in *Mm*Br showed the lowest expression and was presented as one fold.

As the first step towards the characterization of the innate immune competence of different cell lines, the permanent or inducible expression of molecules involved in cell autonomous responses was examined. The PRRs: TLR3, RIG-1 and MDA5, display a various distribution pattern in different cell lines (Fig. 2). Of note, *Mm*Br has the lowest levels of TLR3, RIG-1 and MDA5 (Fig. 2). For TLR3, about 10-fold higher mRNA levels (*p*<0.05) were observed in *Mm*To, *Mm*Pca, *Mm*Nep and *Mm*Nol compared to *Mm*Br, respectively, while for MDA5 about 30-fold (*Mm*To; *Mm*Nep) or about 6-fold (*Mm*Pca; *Mm*Nol) (*p*<0.05) higher expression levels were measured (Fig. 2). Additionally, more than 200 times higher expression levels of RIG-1 were shown in other cell lines compared to *Mm*Br (*p*<0.05) (Fig. 2).

**Figure 3:**
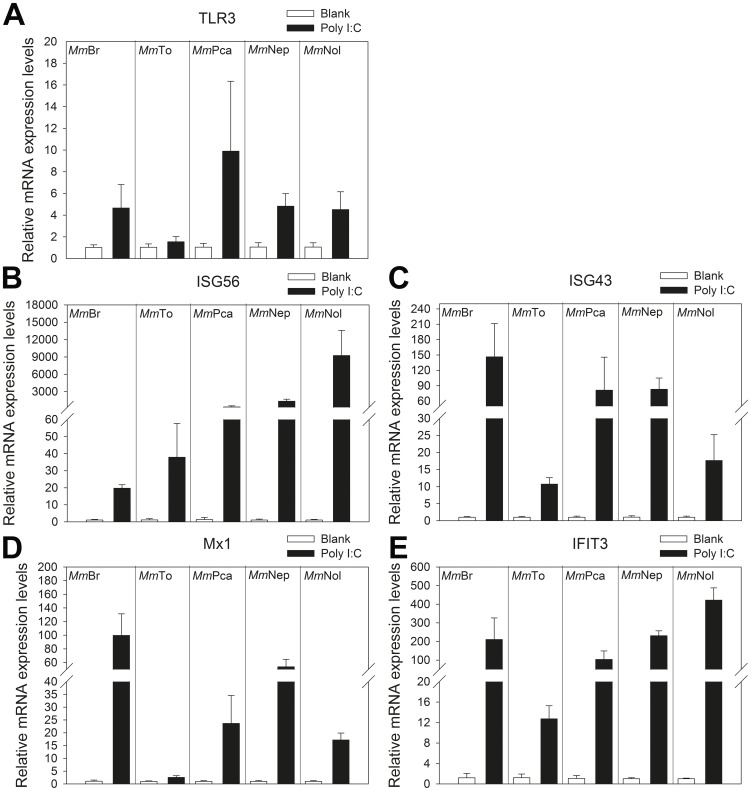
Comparative analysis of the expression patterns of antiviral molecules after poly I:C stimulation. The established immortalized *M. myotis* cell lines were transfected with poly I:C (10 µg/mL) by lipofectamine 2000. The unstimulated cells were used as blank control. Twenty four hours post transfection, the mRNA expression levels of TLR3, ISG56, ISG43, Mx1 and IFIT3 were measured by qRT-PCR (n = 3). The expression level was shown as a related fold and normalized against β-actin. The expression level of different molecules of blank group in individual cell line was presented as one fold.

Further investigation focused on the expression of TLR3, ISG56, ISG43 and Mx1 induced by the poly I:C stimulation (Fig. 3). The obtained results indicate a 4-fold in *Mm*Br, *Mm*Nep and *Mm*Nol and 8-fold in *Mm*Pca (*p*<0.05) increase in the TLR3 expression, whilst no change in *Mm*To (Fig. 3A). All of the IFN induced genes were up-regulated to different extents in different cell lines (Fig. 3B, C, D and E). In detail, ISG56 expression increased from 19-fold in *Mm*Br to as high as more than 9000-fold in *Mm*Nol (*p*<0.05) (Fig. 3B). The expression of ISG43 ranged from 10 to 145 times more and Mx1 from 2 to 100 times more in *Mm*To and *Mm*Br, respectively (Fig. 3C and D). IFIT3 was up-regulated from 12 to 420 times more in *Mm*To and *Mm*Nol, respectively (*p*<0.05) (Fig. 3E).

### *M. myotis* permanent cell lines display different susceptibility to lyssaviruses infection

**Figure 4:**
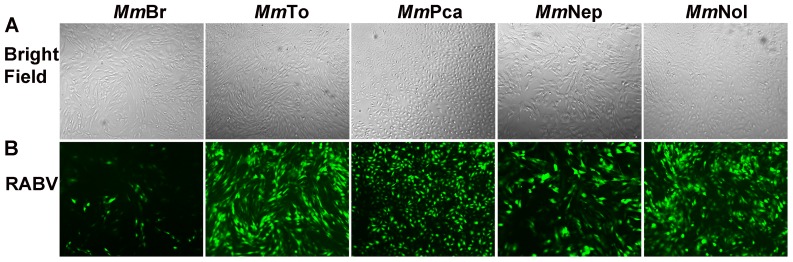
Susceptibility of *M. myotis* cell lines to lyssavirus infection. Immortalized cells (third passage, morphology and cell density visualized in bright filed) were infected with GFP fused RABV at a MOI of 10, and the propagation of RABV was visualized by fluorescence microscope at 24 hpi. Note the low amount of viral antigen positive cells in *Mm*Br in contrast to the other 4 cell lines.

**Figure 5:**
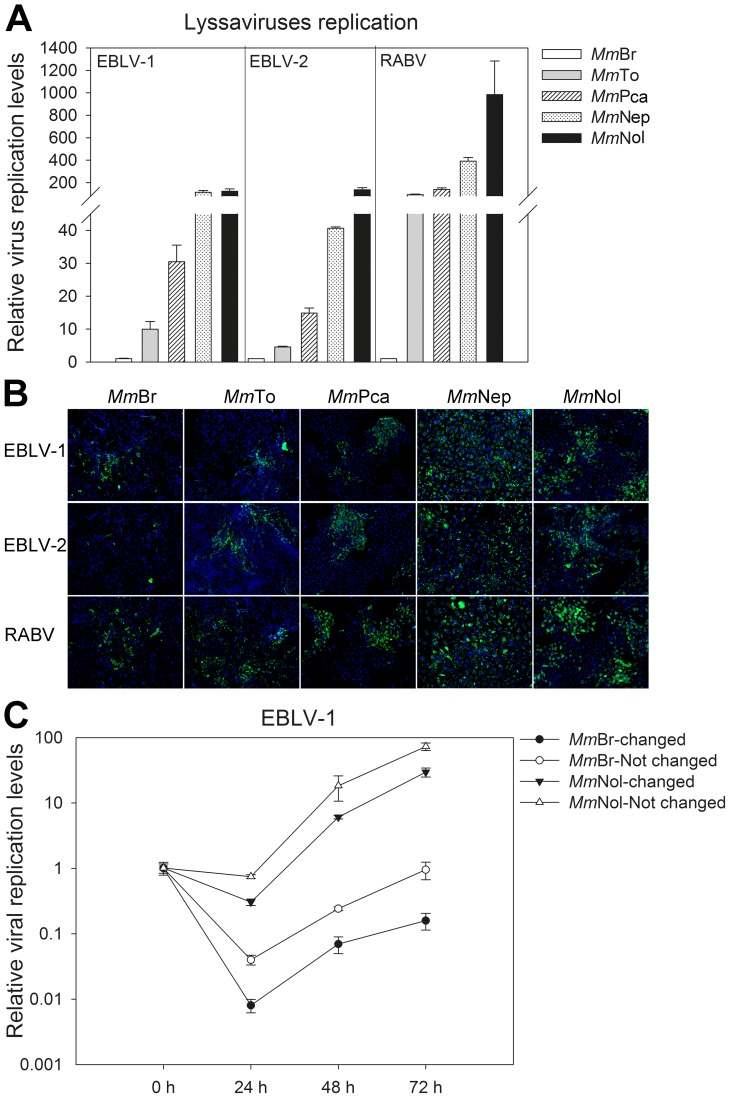
Susceptibility of the established immortalized *M. myotis* cell lines to lyssaviruses (EBLV-1, EBLV-2 and RABV) infection. (**A**) Cell lines were infected with lyssaviruses at a MOI of 0.1, and virus replication levels were measured by qRT-PCR at 24 hpi (n = 2). The viral RNA level was shown as a related fold and normalized against β-actin. The viral replication levels of EBLV-1, EBLV-2 and RABV were lowest in *Mm*Br and presented as one fold, respectively. (**B**) Viral growth was analysed by immunofluorescence using anti-rabies monoclonal antibody at 72 hpi. Green: lyssavirus infected cell, Blue: nuclei stained with DAPI. (**C**) To further confirm the susceptibility, *Mm*Br and *Mm*Nol cell lines were infected with EBLV-1 at a MOI of 0.01, and viral replication levels were measured by qRT-PCR over 72 hpi (n = 2). – changed: viral supernatant was changed with fresh medium at 1 hpi. – Not changed: viral supernatant was not changed during the whole infection process.

Being a natural reservoir species, the main advantage of the permanent *M. myotis* cell lines is their susceptibility to lyssavirus infection. At an early stage of immortalization, cell lines displayed a significant susceptibility to RABV (MOI of 10 at 24 hpi) as demonstrated by the infection with GFP fused RABV. Notably, *Mm*Br exhibited considerably lower viral load compared to the other cell lines (Fig. 4). Later, all passaged immortalized cell lines showed susceptibility to EBLV-1, EBLV-2 and RABV in a different extent (Fig. 5A and B). Generally, the *Mm*Br cell line presented lower sensitivity to all three lyssaviruses (MOI of 0.1) than the other four cell lines measured by qRT-PCR at 24 hpi (Fig. 5A), and monitored by immunofluorescence at 72 hpi (Fig. 5B). Thus, the susceptibility could be ordered as *Mm*Nol and *Mm*Nep fully susceptible with a very high replication rate, *Mm*Pca and *Mm*To susceptible with a much less viral replication of EBLV-1 and 2, *Mm*Br susceptible for EBLV-1 and RABV with a very low viral replication and just single infected cells after EBLV-2 infection (Fig. 5B). The different susceptibility of the cell lines to lyssavirus infection was further confirmed by the growth kinetics of EBLV-1 in two representative models: *Mm*Br, much less susceptible and *Mm*Nol, highly susceptible (Fig. 5C).

### *M. myotis* permanent cell lines respond differently to RABV infection

**Figure 6:**
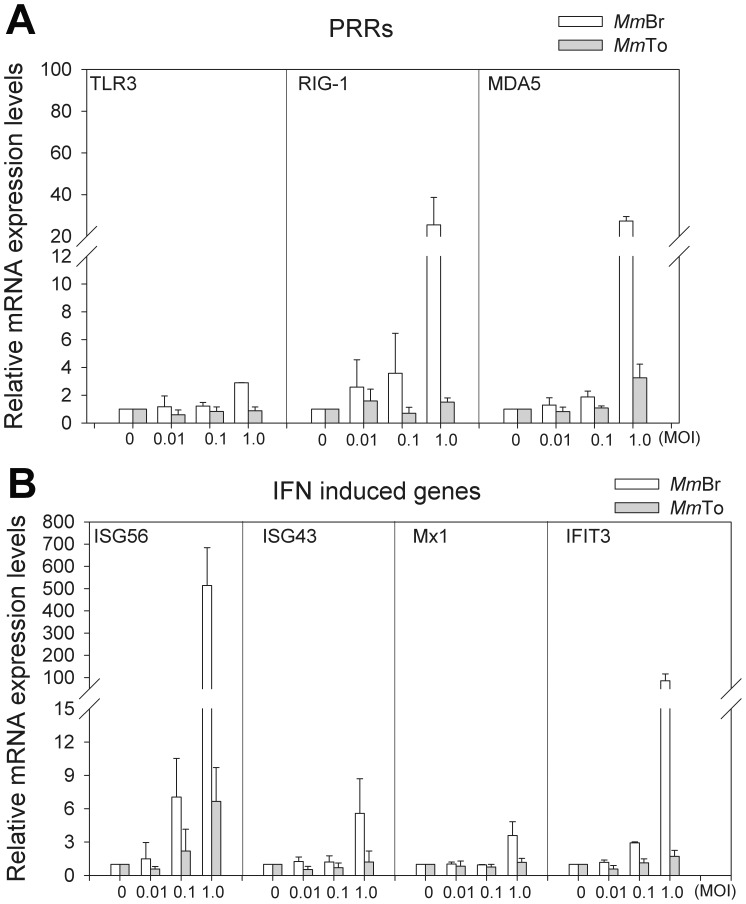
Comparative analysis of the expression patterns of antiviral molecules in *Mm*Br and *Mm*To during RABV infection. *Mm*Br and *Mm*To were infected with RABV at a serial MOI of 0.01, 0.1 and 1.0, respectively. (**A**) The expression patterns of PRRs: TLR3, RIG-1 and MDA5 in the infected cells were investigated by qRT-PCR at 24 hpi (n = 2). (**B**) The expression patterns of IFN induced genes: ISG56, ISG43, Mx1 and IFIT3 were measured by qRT-PCR at 24 hpi (n = 2). The expression level was shown as a related fold and normalized against β-actin. The expression level of different molecules in blank group (MOI: 0) in both cell lines *Mm*Br and *Mm*To was presented as one fold, respectively.

To further evaluate the cell line models for study of the different susceptibility between *Mm*Br and other cell lines, mRNA expressions of PRRs and IFN induced genes were investigated in *Mm*To and *Mm*Br after RABV infection (MOI 0.01 to 1.0). The expression of all three PRRs remained mostly unchanged in *Mm*To, while it was significantly regulated in *Mm*Br with 2-fold increased expression of TLR3, about 25-fold increased expression of RIG-1 and MDA5 at MOI of 1.0 (*p*<0.05) (Fig. 6A). A comparable expression pattern was observed for the ISG56, ISG43, Mx1 and IFIT3, which was nearly not up-regulated in *Mm*To but displayed a dose dependent increase in *Mm*Br along with the increase of MOI, especially for ISG56 and IFIT3 (Fig. 6B). ISG56 mRNA level increased from 6 to 513 times, IFIT3 from 2 to 85 times in the infected *Mm*Br (*p*<0.05).

### The brain derived cell line *Mm*Br are microglia-like cells

**Figure 7:**
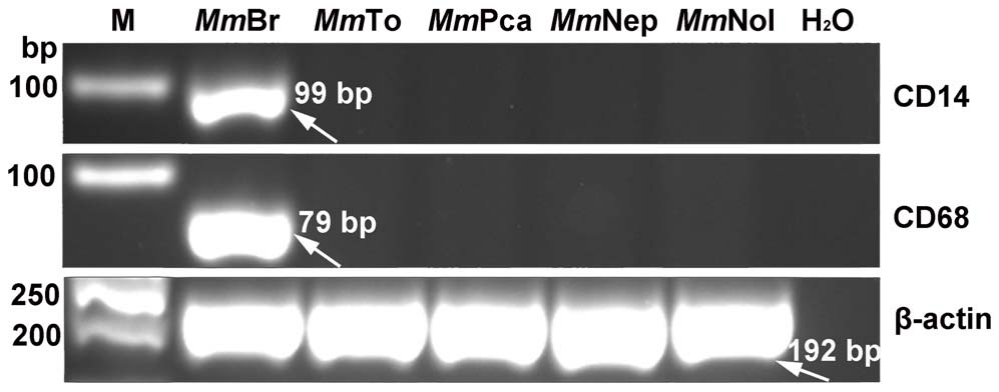
The mRNA expression patterns of CD14 and CD68 in immortalized *M. myotis* cell lines. Note the absence of these two monocyte lineage markers in four of the five cell lines.

Microglia are macrophage-like cells that are resident immune effector cells in the CNS [39]. They are activated in response to infection or injury and play a central role in immune surveillance and host defense [39]. The RT-PCR results showed that CD14 and CD68 are expressed only in *Mm*Br but not in the other four cell lines (Fig. 7). This suggested *Mm*Br is a microglia-derived cell line.

## Discussion

Cell autonomous and innate immune mechanisms are the first line defenses against viral infections. This is mediated mainly by the PRRs and the machinery of the IFNs and IFN induced effector molecules [40]–[42]. Viral pathogens like lyssaviruses developed evasive strategies to escape these host defenses by counteracting the IFN mediated immune responses [43]. Co-evolution of the lyssaviral evading and bat's protective mechanisms resulted in an optimal balance, which protect bats as the ‘natural host’ from severe clinical symptoms or death. Bats, which changed very little over past 52 million years, illustrate this phenomenon very well by the resistance to lethal diseases caused by viruses in other mammals [11], [21]–[24].To understand the specificity of host-pathogen interactions in ‘natural host’ like bats, studies in bats have to be performed. However, due to the strict protection of the endangered European bat species, *in vitro* models have to be used. In this study, we successfully established five *M. myotis* cell lines derived from neural and immune related tissues. To ensure the suitability of these cell lines to analyze virus-host cell interaction, the susceptibility to the infection as well as the presence of corresponding defensive pathways have to be confirmed.

First, the existence of the viral sensors TLR3, RIG-1 and MDA5 in these permanent cell lines suggests a capacity of these cell lines to sense a broad range of RNA viruses. The increased expression of dsRNA receptor TLR3 and IFN induced genes ISG56, ISG43, Mx1 and IFIT3 after stimulation with poly I:C mimicking a viral infection indicates that these cell lines can be used as effective *in vitro* models to study the bat's innate immune responses to virus infection [32], [44]. Furthermore, to serve as valuable models would be a varying susceptibility of such cell lines to infection by lyssaviruses. In the present study, different susceptibility observed in different *M. myotis* cell lines using EBLV-1, EBLV-2 and RABV might be related to the different capacity of the cell lines to produce antiviral mediators and control the infection. Moreover, the strong difference in the susceptibility to RABV infection between *Mm*Br and other cell lines provides a unique opportunity for comparative investigations of cell autonomous and innate immune mechanisms in a reservoir host. In addition to the lyssaviruses, the other member from the *Rhabdoviridae* family, like vesicular stomatitis virus (VSV) can also be investigated by using these models in the future studies. Preliminary results indicate a correlation between the observed varying susceptibility and the ability to up-regulate the PRRs and the IFN induced genes. Emerging evidences have shown that PRRs play pivotal roles in antiviral immunity in the CNS [45]. In the brain derived cell line *Mm*Br, the high up-regulations of RIG-1 and MDA5 revealed activation of RIG-I-like receptor pathway during RABV infection. As previously reported, RIG-1 is a major PRR to induce IFN in the RABV infected cells, and MDA5 may function to sustain the IFN induction [46]. The increased expressions of IFN induced genes: ISG56, ISG43, Mx1 and IFIT3 in *Mm*Br indicate that the production of IFN was induced by activated RIG-1 and MDA5. In contrast, the low expression level of TLR3 implies a vague involvement of TLR3 in anti-RABV infection immunity or resistance. It was shown that TLR3 participated in and benefited the RABV pathogenesis in human neuron cells [47]. However, the roles of TLR3 during RABV infection in bats need further investigations. Importantly, the significant expression patterns of PRRs observed in presented cell line models provide an access to this issue *in vitro*. To reach a successful infection, the viruses must overcome the barriers of innate immune system. It was reported that IFN production and signaling pathways were antagonized in *P. alecto* cell lines under henipavirus infection [32]. Similarly, a recent study showed limited expressions of type I IFNs and IFN induced genes during lyssaviruses infection in an *E. serotinus* brain cell line [33]. A correlation between the low viral load and high expression levels of IFN induced genes in *Mm*Br contrasts to the high viral load and a silent expression pattern of antiviral effectors in *Mm*To, providing an evidence of a countermeasure to IFN system by lyssavirus in the peripheral tissue versus a protective mechanism to infection in the brain tissue of bats. Microglial cells are one of the major cell populations in the brain tissue. Additionally, comparing to neurons, they can be infected by different RABV strains to a lesser extent [48], [49]. The presence of CD14 and CD68 as well as the anti-lyssavirus responses in *Mm*Br support a microglia-like feature of *Mm*Br in the CNS. It was reported that a mouse microglia cell line can activate strong innate immunity during RABV infection [50]. The robust immune responses of the microglia-like *Mm*Br demonstrated a critical role of microglia in the anti-rabies defense in bat's CNS. In addition to the function of microglia, the clearance of infected viruses in the CNS requires systematical responses through the complex interactions of different brain resident cells. Herein, the establishment and identification of a microglia-like cell model is a first step towards understanding of the complex reactions of CNS in response to lyssavirus infection in the reservoir species. Overall, this preliminary study using established cell lines implies that immune mechanisms that control the virus replication are present in the CNS of bats. It seems that the ability to control the pathogenic RABV replication via IFN system in the CNS contributes to the asymptomatic outcome in bats.

In conclusion, the established immortalized cell lines from the European bat *M. myotis* displaying a variable susceptibility to different lyssaviruses will serve as a useful model to study virus-host interactions and antiviral resistance mechanisms in the ‘natural’ *Lyssavirus* host. This study provides a preliminary insight into the antiviral innate immunity correlated to CNS against neurotropic viruses infection in bats.
